# Middle cranial fossa tumor presenting as chronic otitis media: Rare case of aneurysmal bone cyst

**DOI:** 10.1016/j.ijscr.2023.108996

**Published:** 2023-10-27

**Authors:** Chia-Fang Shen, Chin-Chan Yang, Szu-Yuan Liu, Chiung-Chyi Shen

**Affiliations:** Department of Neurosurgery, Neurological Institute, Taichung Veterans General Hospital, 1650 Taiwan Boulevard Sect. 4, Taichung City 40705, Taiwan, ROC

**Keywords:** Aneurysmal bone cyst, Chronic otitis media, Middle cranial fossa tumor, Case report

## Abstract

**Introduction:**

Aneurysmal bone cysts (ABCs) are a rare, vascular, rapidly growing, benign, osteolytic lesions. Most ABCs involve the metaphysis of long bones, vertebrae, or flat bones. In this study, we review the literature to better understand the natural history, clinical presentation, and treatments.

**Presentation of case:**

A 34-year-old man who presented with left intermittent otorrhea for months. Yellowish, pus-like discharge was noted. Mild tinnitus, hearing loss, and occasional headache was also found. The initial impression was chronic otitis media and ear drops were prescribed. However, his symptoms did not improve in the following months. The brain MRI with gadolinium enhancement revealed an extra-axial mixed signal intensity lesion on the T2-weighted image, multiloculated cystic components and rim enhancement was noted over the left middle cranial fossa. Left fronto-temporal craniotomy for tumor removal was performed. The pathological reports revealed an aneurysmal bone cyst.

**Clinical discussion:**

Typically, ABCs present with localized swelling and pain due to their rapid growth and expansion, with concomitant signs corresponding with the anatomical location of the lesion. MRI studies can reveal the cystic components of the lesion and multiple fluid levels within multiloculated cysts resulting from unclotted blood, separated from the soft tissue and medullary bone. Histopathologic diagnosis of ABC is the presence of multiple blood-filled cystic spaces separated by fibrous septa. The fibrous septa are composed of spindle-celled fibroconnective tissue with occasional osteoclast-type giant cells.

**Conclusion:**

ABCs are a rare, osteolytic lesions that rarely involve the skull. When the MRI shows a lesion with soap-bubble appearance in the calvaria, an aneurysmal bone cyst should be considered in the differential diagnosis, even if it is an extremely rare entity or the patient is relatively old. Surgical resection of the tumor is the first choice for treatment.

## Highlights

• Aneurysmal bone cysts are rare, vascular, rapidly growing, benign, osteolytic lesions that rarely involve the skull.
• Preoperative diagnosis of ABCs from another osteolytic lesion of the cranium is difficult. However, MRI shows the characteristics of the tumor, which include a multiloculated, expansile bony lesion with fluid–fluid levels in one or more granular spaces and a hypointense circular rim surrounding the entire lesion, an aneurysmal bone cyst should be considered, even if it is an extremely rare entity or the patient is relatively old.
• Complete surgical resection of the tumor is the first choice for treatment if its location is assessed. Possible reconstruction after resection and postoperative adjunctive therapy for the complex lesion is an important issue to prevent tumor recurrence and restore neurological function.

## 1 Introduction and importance

Aneurysmal bone cysts (ABCs) are a rare, vascular, rapidly growing, benign, osteolytic lesions that were first described by Jaffe and Lichtenstein in 1942 [1]. They consist of multiple thin-walled, blood-filled cystic cavernous cavities without endothelial cells. Most ABCs involve the metaphysis of long bones, vertebrae, or flat bones and typically occurs before the age of 20 years. Occasionally, ABCs are found in the cranium, as described in 3 %–6 % of cases [1]. The etiology and pathophysiology of ABCs remain uncertain, but it is assumed that factors such as trauma, underlying neoplasms, and cytogenetic abnormalities are thought to be the possible pathophysiology of ABCs [9]. In this study, we report a rare case of an intracranial aneurysmal bone cyst presenting as chronic otitis media, we also review the literature in order to better understand and categorize the natural history, clinical presentation, imaging features and treatments. This case report has been reported in line with the SCARE 2020 criteria [2].

## 2 Case presentation

A 34-year-old man who had no systemic disease presented with left intermittent otorrhea with since June 2022. Yellowish, pus-like discharge was noted. In addition, mild tinnitus, hearing loss, and occasional headache was reported by the patient. There was no complaint of fever, dizziness, or ear pain. The initial impression was chronic otitis media and antibiotic ear drops were prescribed. However, his symptoms did not improve in the following month. Then, one evening after work in September 2022, he suffered from acute onset of vertigo and unsteady gait. He denied trauma and the symptoms lasted several hours. Moreover, his symptoms could not be relieved by changing position. Thus, he was sent to the emergency room the next day. At the ER, the physical examination showed normal extraocular movement (EOM), but horizontal nystagmus was noted. Otolaryngologist was consulted and fiberscope showed redness with scaling at the left inner two-thirds of the external auditory canal (EAC). Tympanic membrane (TM) thickening with brownish pus coating and turbid content behind the tympanic membrane were observed. Left eardrum effusion indicated the possibility of a cyst or otitis media with effusion (OME). Pure tone audiometry (PTA) revealed mixed type hearing loss, with an air-bone gap of 20–30 dB.

**Fig. 1:**
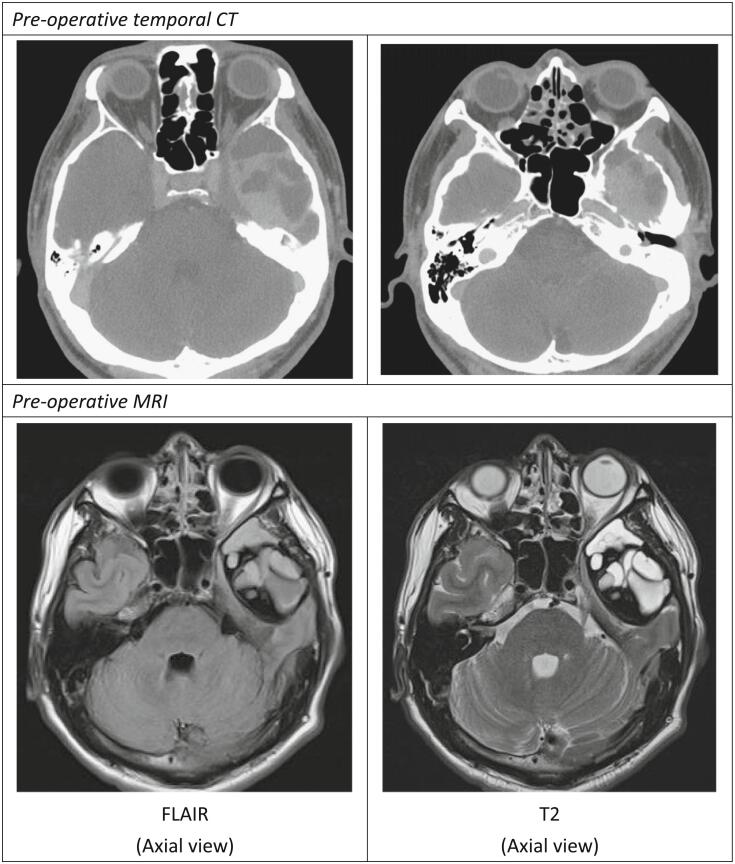

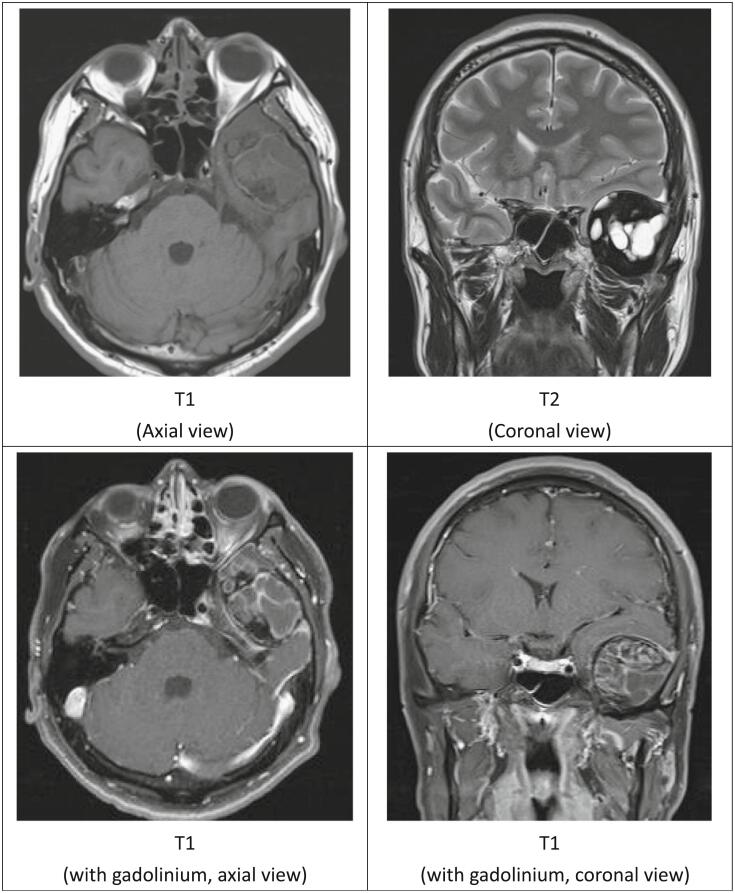
Pre-operative image.

Imaging studies were arranged for evaluation, including computed tomography (CT) and magnetic resonance imaging (MRI) [Fig. 1]. Temporal CT demonstrated a well-defined mass lesion, measuring about 5.6 cm at the left middle cranial fossa with enhanced solid part and non-enhanced cystic part, with bony erosion of adjacent skull base connection to the left middle ear cavity, ruling out cholesteatoma or other skull base tumor with invasion. The results of brain MRI with gadolinium enhancement revealed an extra-axial mixed signal intensity lesion on the T2-weighted image and fluid-attenuated inversion recovery (FLAIR) with multiloculated cystic components and rim enhancement was noted over the left middle cranial fossa. Origin from skull base was favored, with differential diagnosis including aneurysmal bone cyst with internal hemorrhage, neurogenic tumor, and pigmented villonodular synovitis.

**Fig. 2:**
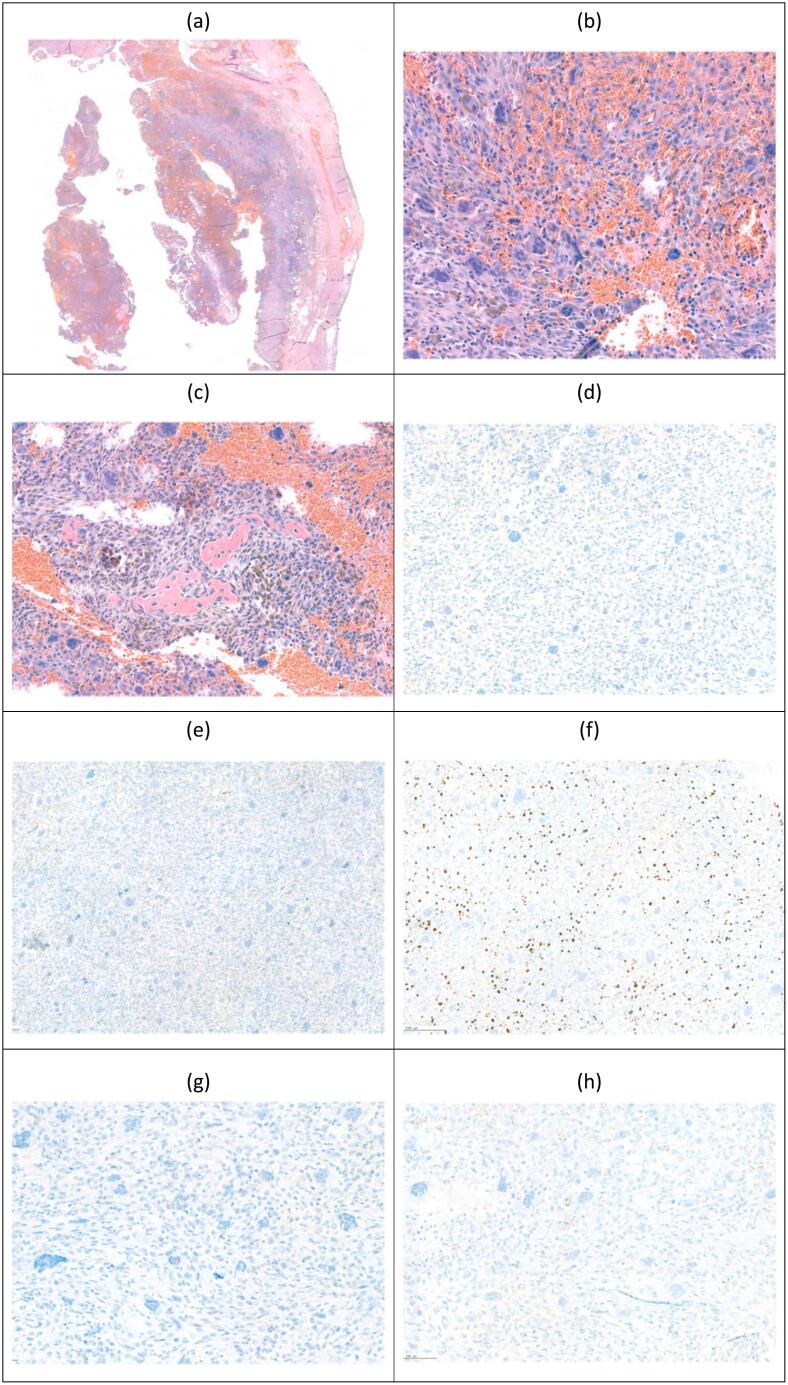
Histopathologic features of aneurysmal bone cyst. [a: 500um, b: 100um, c: 100um] A blood-filled cystic spaces with cystic wall composed of spindle cells with osteoclast-like giant cells and reactive woven bone. Some inflammatory cells and hemosiderin deposition were found. Immunohistochemical (IHC) studies: [d: 250 μm, e: 500 μm, f: 500 μm, g: 200 μm, h: 200 μm]. Negative for (d) Epithelial membrane antigen (EMA), (e) H3.3 G34W (specific protein mutation), (f) Ki-67 (weak expression) (g) Progesterone receptor (PR) (h) Signal transducer and activator of transcription 6 (STAT6).

Left fronto-temporal (mini-pterional) craniotomy for tumor removal was performed in October 2022. Intraoperative findings showed a well-defined extradural lesion at the left temporal base with severe adhesion to the dura. It was composed of multiple cysts filled with yellowish fluid. Moreover, the tumor was soft and rich in vessels, but there was no gross brain parenchyma invasion. Gross total resection of the tumor was performed under microscope. Bone erosion of the temporal base was found after tumor removal. A piece of dura which had adhered to the tumor was also removed and duraplasty with artificial dura (Duragen-Plus) was done. We used Tisseel solution, Geofoam for hemostasis, and prevented CSF leakage after duraplasty. The pathological reports revealed an aneurysmal bone cyst. A blood-filled cystic space was noted with a cystic wall composed of spindle cells with osteoclast-like giant cells and reactive woven bone. Immunohistochemistry stains were done for epithelial membrane antigen (EMA), progesterone receptor (PR), signal transducer and activator of transcription 6 (STAT6), glial fibrillary acidic protein (GFAP), H3.3 G34W, p63 was negative, and ki-67 (weak expression) [Fig. 2].

**Fig. 3:**
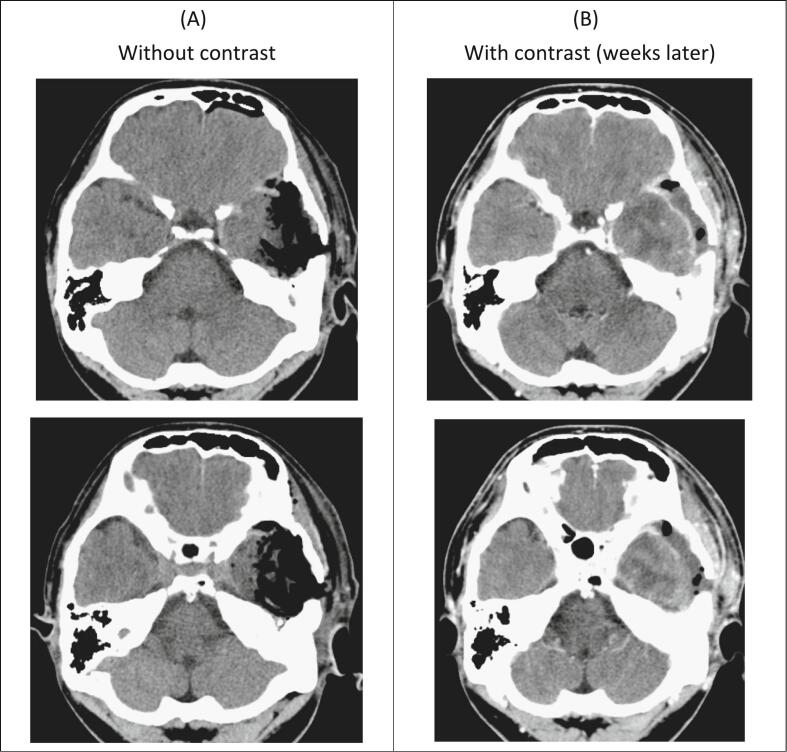
Post-operative CT.

The follow-up non-contrast-enhanced brain CT is shown in Fig. 3. The patient recovered well and symptoms, such as dizziness, otorrhea, and imbalanced gait, were much improved after the operation. However, about three weeks after the operation, the patient complained of left hemifacial numbness. Left ear fullness sensation and impaired hearing also developed again. Left eardrum effusion was found by an otolaryngologist. Intermittent severe headache was subsequently reported by the patient at the occipital region, with bouts lasting for 1–2 min. This pain was sharp, and sometimes migrated to the frontal region. In addition, fever up to 38.4′C with chillness developed. Neurological examination showed clear consciousness, intact orientation, with GCS of E4V5M6, and full muscle power. Laboratory data revealed leukocytosis (WBC count: 14880) with mildly elevated level of CRP (0.336). Cerebrospinal fluid (CSF) was collected by lumbar puncture, and results showed the TNC (Total Nucleated Count) 3540 /uL with neutrophil predominance (88 %). Further image survey with contrast-enhanced brain CT was performed and it disclosed increased leptomeningeal enhancement at the bilateral fronto-parietal region and extra-axial fluid, as well as air collection at the left temporal region [Fig. 3]. Postoperative change or possible infectious meningitis with epidural abscess could not be ruled out. No tenderness, pus-like discharge, or erythematous change over the operative wound, except mild swelling, were noted.

Under the impression of meningitis, antibiotic treatments with vancomycin and ceftriaxone were prescribed, which were then changed to linezolid and ceftriaxone due to allergy to vancomycin. Culture studies revealed no pathogen growth in samples of discharge and CSF, but the middle ear effusion showed *Klebsiella aerogenes*. Furthermore, the swelling wound had turned hard and had started to suppurate. We performed a combined surgery for debridement and possible CSF leakage. Epidural abscess at the temporal region and a mass-like lesion at the left EAC with some discharge were noted. No skull bone defect or CSF leakage was found. The pathological report revealed a granulation tissue with chronic active inflammation. The patient recovered well after finishing a course of antibiotics.

## 3 Clinical discussion

Aneurysmal bone cysts (ABCs) are rare, vascular, benign, expansible, osteolytic lesions that were first described by Jaffe and Lichtenstein in 1942. Jaffe and Lichtenstein coined the term aneurysmal bone cyst due to a specific entity consisting of an expanding bony lesion with a vascular lining and a soap-bubble radiologic appearance [1]. These blood-filled spaces were separated by connective tissue septa composed of bone or osteoid and osteoclast giant cells [3]. They could affect any bone, including skull bone, but they are most commonly found in the metaphysis of long bones, vertebrae, or flat bones. Most cases occur in the second decade of life without gender predominance [1,3].

### 3.1 Origin of ABCs

The origin and nature of ABCs remains unclear, though there are several theories that have been proposed to explain its origin. According to one theory, ABCs develop due to a reactive process following a trauma that induces a local circulatory disturbance. An abnormally increased venous pressure leads to hemorrhage, with subsequent osteolysis. A phase of active growth follows initial osteolysis, after which maturation and ultimately bony healing occur. This osteolytic lesion formation promotes additional hemorrhages and, in turn, results in the amplification of the developing cyst [4]. In another theory, ABCs are associated with unique cytogenetic abnormalities. Approximately 50 % of patients with these lesions show an abnormality on chromosome 16q or 17p (16q22, 17p11–13). A chromosomal translocation that causes the upregulation of ubiquitin-specific proteases leads to increased cell adhesion. It has been associated with solid variant and extraosseous forms, which supports the theory that ABCs are true neoplasms [5]. A third theory posits that ABCs arise from an underlying neoplasm that induces a venous obstruction or an arteriovenous fistula [6]. A current and popular view is that these lesions were classified as primary and secondary depending on the presence or absence of associated pathology, the most common of which are giant cell tumors of bone, chondroblastomas, osteoblastomas, osteogenic sarcomas, chondromyxoid fibromas, and fibrous dysplasia [3,7]. Hormonal influence on the growth of these lesions has also been proposed, especially during pregnancy. Its association with pregnancy and rapid enlargement of the lesion during pregnancy, though rare, have also been reported [8].

### 3.2 Clinical presentation and imaging findings

Typically, ABCs present with localized swelling and pain due to their rapid growth and expansion, with concomitant signs corresponding with the anatomical location of the lesion [3,7,9]. The most common symptom is pain at the site of the lesion. In the cranium, ABCs can present with headache, vertigo, tinnitus [16], exophthalmos, ptosis, visual impairment [7], otorrhea [14], cranial nerve palsies [19], seizure disorder [18], cerebellar signs [10], and signs of raised intracranial pressure [11,16,17]. Bacterial meningitis has also been described in the previous studies [12].

Plain radiographs of aneurysmal bone cysts in the skull typically reveal an eccentric, ballooned, cystic expansion surrounded by a sclerotic rim [20]. The periosteum may be raised by new bone formation growing between the margin of the ABC and the adjacent, unaffected bony cortices. Plain films demonstrate either single or multiple blown-out lesions in the bone, hence the term aneurysmal [13]. On CT scans, well-demarcated, multiloculated, osteolytic lesions have a soap-bubble appearance. The multiloculated lesion typically consists of several cavities with different densities. The vascular stroma and septa may enhance peripherally after administration of the contrast material [10]. In addition, fluid–fluid levels are sometimes apparent on a CT scan when the patient is immobile for a few minutes.

MRI studies can reveal the cystic components of the lesion and multiple fluid levels within multiloculated cysts resulting from unclotted blood, separated from the soft tissue and medullary bone. A low signal intensity rim surrounding the entire lesion correlates with the pathologic finding of a fibrous capsule. Fluid-fluid levels are an important cross-sectional imaging finding on MRI, but they are nonspecific and are more easily seen on an MRI than on a CT scan. The cyst contents consist of blood degradation products in different stages of evolution and, as such, the signal intensity of the degradation products varies, a finding considered to be attributable to methemoglobin [13]. Other bone lesions with bleeding in the cystic component may also show fluid–fluid levels on MRI, such as giant-cell tumor, chondroblastoma, telangiectatic osteosarcoma, and malignant fibrous histiocytoma [14].

On angiograms, the features of cysts elsewhere in the body are usually characterized by a pathological circulation with a patchy distribution of contrast, as well as persistent venous circulation and occasional arteriovenous shunting. This finding may be absent when the lesion is located in the cranium, indicating that it is an avascular mass lesion [10].

### 3.3 Histopathology

Histological characteristics of ABCs are well defined and histologic evaluation is imperative for definitive diagnosis. Grossly, ABC is a relatively well-circumscribed, friable, multicystic, and hemorrhagic lesion [15]. Histopathologic diagnosis of ABC is based on the presence of multiple blood-filled cystic spaces separated by fibrous septa. The fibrous septa are composed of spindle-celled fibroconnective tissue with occasional osteoclast-type giant cells. Furthermore, inflammatory cells are common, and immature bone formation can also be identified, often along the fibrous septa. Generally, atypical mitoses are not seen [15].

### 3.4 Treatments

Although ABCs have no malignant or metastatic potential, local mass effect and alteration of locoregional vascularity may be severe, necessitating treatment [16], and treatment for primitive ABCs depends on the patient's age, location and size of the lesion. Options include surgical resection, endovascular embolization, radiotherapy, sclerotherapy, or cryosurgery [6,17]. Surgical excision with a wide margin of the lesion is the most accepted treatment option and is usually curative, if the lesion can be easily accessed and can be completely excised [16]. However, complete surgical resection may be more difficult to achieve when the lesion is large or when it involves the skull base. When a complex condition such as this is encountered, partial excision or intralesional curettage with adjunctive therapy should be considered, such as preoperative embolization, postoperative radiotherapy, or cryotherapy [17]. Reconstruction for these difficult-to-access lesions after surgical excision is also an important topic, and combined surgery is sometimes necessary. Preoperative embolization also reduces bleeding during surgery. Radiotherapy is not recommended because of the risk of sarcomatous degeneration [17]. However, the use of radiotherapy is mentioned in the literature and has been advocated for deeply situated lesions at the base of the skull with dura involvement, where subtotal excision is done, though its effect is unclear.

## 4 Conclusion

Aneurysmal bone cysts are rare, vascular, rapidly growing, benign, osteolytic lesions that rarely involve the skull. Preoperative diagnosis of ABCs from another osteolytic lesion of the cranium is difficult. However, MRI shows the characteristics of the tumor, which include a multiloculated, expansile bony lesion with fluid–fluid levels in one or more granular spaces and a hypointense circular rim surrounding the entire lesion. When a lesion with soap-bubble appearance and fluid–fluid levels is found in the calvaria, an aneurysmal bone cyst should be considered in the differential diagnosis, even if it is an extremely rare entity or the patient is relatively old. Complete surgical resection of the tumor is the first choice for treatment if its location is assessed. Possible reconstruction after resection and postoperative adjunctive therapy for the complex lesion is an important issue to prevent tumor recurrence and restore neurological function. The patient described herein completely recovered, though complications with meningitis occurred during the postoperative course.

## Consent

Written informed consent was obtained from the patient for publication of this case report and accompanying images. A copy of the written consent is available for review by the Editor-in-Chief of this journal on request.

## Provenance and peer review

Not commissioned, internally reviewed.

## Ethical approval

Not required for case reports at our hospital. Single case reports are exempt from ethical approval in our institution.

## Funding

This research did not receive any funding agencies in the public, commercial, or not-for-profit sectors.

## Author contribution

**Chia-Fang Shen**: Operation, Conceptualization, Methodology, Validation, Investigation, Resources, Data Curation, Writing - Original Draft, Review & Editing, Visualization, Project administration.

**Chin-Chan Yang**: Operation, Conceptualization, Methodology, Validation, Formal analysis, Writing - Original Draft.

**Szu-Yuan Liu**: Operation, Software, Validation, Formal analysis, Investigation, Resources, Data Curation, Writing - Review & Editing, Visualization, Supervision.

**Chiung-Chyi Shen**: Visualization, Supervision.

## Guarantor

Chiung-Chyi Shen– Chief of Neurological Institute, Head of Department of Neurosurgery, Taichung Veterans General Hospital, 1650, Taiwan Boulevard Section 4, Taichung, Taiwan 407, ROC.

## Research registration number

Not applicable.

## Conflict of interest statement

All authors declare that they have no competing interests.
